# The Role of Adipokines in Intervertebral Disc Degeneration

**DOI:** 10.3390/medsci6020034

**Published:** 2018-04-24

**Authors:** Anirudh Sharma

**Affiliations:** Division of Orthopaedic Surgery, University of Manitoba, Winnipeg, MB R3A 1R9, Canada; sharma39@myumanitoba.ca; Tel.: +1-204-787-7581

**Keywords:** disc degeneration, adipokines, leptin, adiponectin, resistin, spine, obesity

## Abstract

Intervertebral disc degeneration (IDD) is an important cause of low back pain. Recent evidence suggests that in addition to abnormal and excessive mechanical loading, inflammation may be a key driver for both IDD and low back pain. Obesity, a known mechanical risk factor of IDD, is now increasingly being recognized as a systemic inflammatory state with adipokines being postulated as likely inflammatory mediators. The aim of this review was to summarize the current literature regarding the inflammatory role of adipokines in the pathophysiology of IDD. A systematic literature search was performed using the OVID Medline, EMBASE and PubMed databases to identify all studies assessing IDD and adipokines. Fifteen studies were included in the present review. Leptin was the most commonly assessed adipokine. Ten of 15 studies were conducted in humans; three in rats and two in both humans and rats. Studies focused on a variety of topics ranging from receptor identification, pathway analysis, genetic associations, and proteonomics. Currently, data from both human and animal experiments demonstrate significant effects of leptin and adiponectin on the internal milieu of intervertebral discs. However, future studies are needed to determine the molecular pathway relationships between adipokines in the pathophysiology of IDD as avenues for future therapeutic targets.

## 1. Introduction

**Figure 1:**
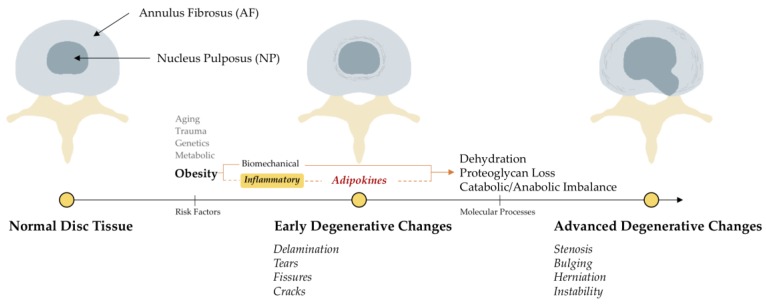
The anatomy and pathophysiology of intervertebral disc disease. The dotted line represents the focus of the present review.

Intervertebral disc degeneration (IDD), an important cause of low back pain (LBP), has traditionally been considered as an age-related process of ‘drying and cracking’ of the disc tissue caused by decreased proteoglycan content, leading to the classic radiological signs of decreased intervertebral height, end-plate sclerosis and osteophytosis [1,2]. The normal human intervertebral disc is a fibrocartilaginous structure consisting of two parts: (i) the outer fibrous annulus composed of fibroblast-like cells and collagen type I; and (ii) the inner soft nucleus composed of chondrocyte-like cells, proteoglycan and water [3,4,5]. This proteoglycan loss begins early in life but factors such as aging, altered mechanical loading, trauma, metabolic disturbances and unfavorable genetics can hasten disc weakening causing physical disruption, ultimately leading to the degenerative pathology [6] (Figure 1). While many patients with radiological evidence of IDD remain asymptomatic [7], some exhibit clinical symptoms of LBP. Initial management of these symptomatic patients includes conservative measures such as back exercises, pain killers, anti-inflammatories and/or injections. Since these measures do not address the underlying etiology of disc degeneration, disease progression and/or development of neurological symptoms often necessitate the need for back surgery in some [8]. These symptomatic IDD patients often report major disability, decreased quality of life and high individual and societal economic burden [9].

Of the many factors responsible for IDD, obesity has largely been implicated as a mechanical risk factor in the course of disc disease [10,11]. However, the literature suggests that its role has been a topic of debate. While some studies have reported a significant positive association between obesity and IDD [11,12], others have found none [13,14,15]. These disparities in reporting cannot be solely explained by aging or mechanical effects of obesity. In addition to its mechanical effects, obesity also exerts metabolic/inflammatory effects [16,17]. Adipose tissue is now being recognized as an active organ with numerous metabolic/inflammatory properties. Adipocytes secrete specific distinct proteins called adipokines, which can either act locally or reach distant tissues via systemic circulation and exert either pro- or anti-inflammatory effects [18]. Leptin, resistin, chemerin, retinol binding protein 4 (RBP4), and lipocalin 2 (LCN2) exhibit pro-inflammatory properties, while adiponectin, C1q/tumor necrosis factor (TNF)-related proteins (CTRPs), omentin and secreted frizzled-related protein 5 (SFRP5) are primarily anti-inflammatory [18,19]. Most adipokines carry out their effects via binding to cellular receptors causing activation of intracellular protein pathways.

Outside of IDD, these adipokines have consistently been reported to play an important role in musculoskeletal diseases like osteoarthritis (OA) [19,20,21,22,23], metabolic diseases like diabetes [14] and cardiac diseases like hypertension and atherosclerosis [24]. They exert direct effects on chondrocyte function, glucose homeostasis, and endothelial integrity. Recent evidence suggests that inflammation may play a role in the development of IDD [25,26,27]. However, whether adipokines act as mediators remains largely unknown. Therefore, the aim of the present review is to provide a systematic overview of the role of adipokines in the pathophysiology of IDD, reflecting on various signaling pathways that could be manipulated as potential future therapeutic targets.

## 2. Materials and Methods

### 2.1. Eligibility Criteria

Original studies assessing (1) disc degeneration and (2) adipokines were included in this present review. Review articles, letters to the editor, published abstracts, editorials and case studies or reports were excluded. Additionally, only articles with full-text versions published in the English language were eligible for this review.

### 2.2. Search Strategy and Criteria

A manual electronic search of the OVID Medline (from 1946), EMBASE (from 1974) and PubMed databases, encompassing all articles until 2 April 2018, was performed in separate to identify all studies assessing IDD and adipokines. The following search string was used: “((adipokines OR leptin OR adiponectin OR resistin OR adipsin OR visfatin) AND (intervertebral disc OR disc degeneration))”. Seventy records were initially identified with 38 remaining after performing de-duplication. These records were manually screened for eligibility following which three non-English records were excluded. The remaining 35 records were assessed for inclusion in the final review.

### 2.3. Study Selection

**Figure 2:**
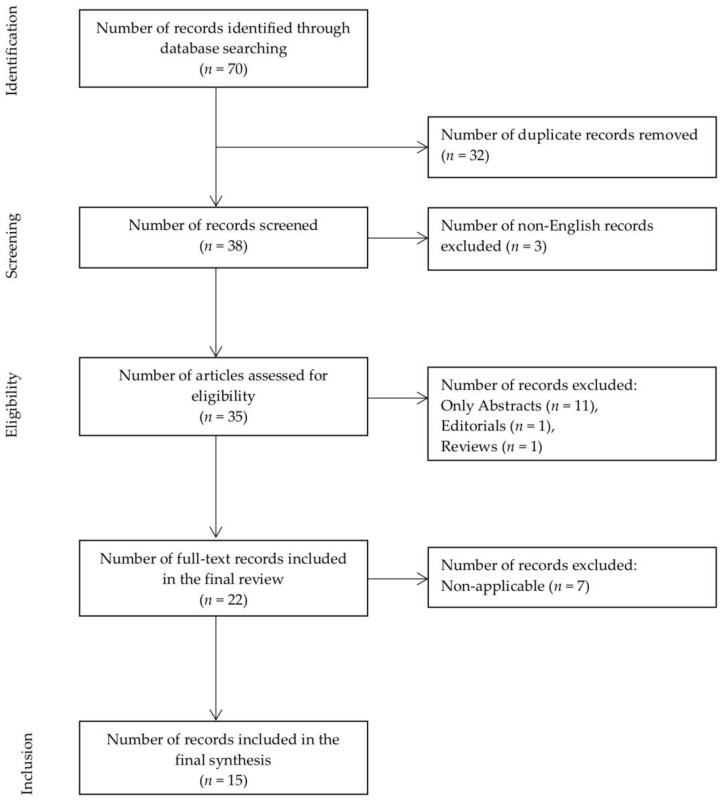
Flow diagram summarizing the literature search, screening and review.

Complete citations of the remaining 35 articles were extracted to Excel spreadsheet and sorted by publication-type metadata. Publication types not meeting the inclusion criteria were discarded. After excluding 11 abstracts, one editorial, one review and seven records based on title, abstract and publication information; 15 records were included in the final review (Figure 2, Table 1) [28,29,30,31,32,33,34,35,36,37,38,39,40,41,42]. Full-length articles of the remaining 15 records were obtained and screened. All the final studies included in this review were experimental laboratory studies with well-defined hypotheses.

### 2.4. Data Collection

Data extraction included the following elements: (1) study authors (2) year of publication (3) study design and (4) key findings (5) Sample.

## 3. Results

From 70 citations initially identified, 15 were considered potentially relevant and their full manuscripts were evaluated. All studies were published from 2007 onwards and included age groups ranging from young [29,35], adult [28,30,32,33,34,35,37] to elderly [31] cohorts. All were in vitro studies focusing on a range of topics from receptor identification [29,37], pathway analysis [28,32,34,36,38,40,41,42], genetic associations [30] and proteonomics [31,33,37]. Four human studies compared results between cases and controls [29,30,31,39].

### 3.1. Adipokines

Leptin was the most commonly assessed adipokine. Ten of 15 studies evaluated leptin and/or their receptors via utilizing different methodologies ranging from but not limited to Western blot (WB), immunohistochemistry (IHC), polymerase chain reaction (PCR), and enzyme-linked immunosorbent assay (ELISA) [28,29,31,32,33,34,35,36,37,38]. Additionally, three studies assessed adiponectin [30,39,40], while two studies assessed resistin [41,42].

### 3.2. Sample/Tissue

Eleven of 15 studies (seven human [29,30,31,32,33,34,35]; two animal [28,36] and two both [37,40]) studied nucleus of the intervertebral disc tissue [32,33,34,35,36,37,38,39,40,41,42], whereas three (one animal [28] and two human [29,31]) studies evaluated annulus of the intervertebral disc tissue [28,29,31]. The remaining one study only tested plasma samples [30].

### 3.3. Species

Ten of 15 studies were conducted in humans [29,30,31,32,33,34,35,38,39,42], three studies were conducted in rats [28,36,41] and two studies were conducted in both humans and rats [37,40].

#### 3.3.1. Human Studies

On studies focusing on human subjects, Gruber et al. and Zhao et al. reported the presence of leptin and leptin receptors in annulus [29] and nucleus [37] cells. Likewise, Koerner et al. reported higher expression of cytokines and growth factors in the posterior annulus compared with the anterior annulus in patients with IDD [31]. On examining the effects of leptin on cultured nucleus cells Li et al. reported that leptin: (i) induced cyclin D1 expression and proliferation via activation of JAK/STAT3, PI3K/Akt or MEK/ERK signaling [33]; (ii) induced p38 to upregulate a disintegrin and metalloproteinase with thrombospondin motifs (ADAMTSs), thereby promoting aggrecan degradation [35]; (iii) activated the RhoA/ROCK/LIMK/cofilin-2 cascade to induce cytoskeleton reorganization [32]; (iv) and increased expression of beta-actin, vimentin and reorganization of F-actin [34]. The authors additionally reported that mRNA and proteins of OBRa and OBRb were expressed in all nucleus tissues and cells, and OBRb expression was correlated with body weight [34]. Similarly, Zhang et al. reported that leptin-treated cells showed increased expressions of LC3II/I and Beclin-1 and decreased apoptosis rate [38]. Furthermore, the authors reported that inhibition of Akt phosphorylation was partially offset by leptin while inhibition of Erk1/2 phosphorylation was not.

Yuan et al. examined the expression levels and effect of adiponectin on TNF-α in disc tissues and isolated nucleus cells and concluded that adiponectin levels were downregulated, while AdipoR1 and AdipoR2 receptor expression was upregulated in degenerated disc tissue and nucleus cells compared to healthy controls [39]. They also reported that TNF-α production by degenerated nucleus cells was downregulated by adiponectin administration. Conversely, in a study by Khabour et al., the authors reported that plasma adiponectin levels were elevated in patients with lumbar disc degeneration [30].

#### 3.3.2. Animal Studies

On studying the action of leptin, Zhao et al. reported that leptin stimulated proliferation of disc cells in vitro [37]. Similarly, Miao et al. reported that leptin promoted catabolic metabolism in the nucleus cells via the MAPK and JAK2/STAT3 pathways, which could be a mechanism mediating the association between obesity and IDD [36]. Likewise, Ding et al. concluded that MAPK pathway (p38 and ERK1/2 signaling) played a distinct role in leptin-induced annulus cells terminal differentiation [28]. In a parallel animal experiment conducted by Terashima et al., the authors reported higher expression of AdipoR1 and AdipoR2 receptors in both human and rat disc tissue, which was inversely related to disease severity [40]. Furthermore, the authors found TNF-α expression in the IL-1β + Adiponectin group to be significantly lower compared to IL-1β group, in both the nucleus and annulus cells.

## 4. Discussion

Low back pain is a pressing health condition worldwide with lifetime prevalence as high as 84% [43]. It is one of the leading causes of physical disability and loss of work days, imposing a high socioeconomic burden [44,45]. Eight in 10 adults will experience low back pain at some point in their lifetime [46]. These alarming results suggest a considerable gap in our basic understanding and management of the disease; thus, challenging us to focus our research efforts in expanding our knowledge in finding new avenues of preventive and therapeutic targets. Intervertebral disc degeneration is frequently linked as a critical source for low back pain [2,47]. Nevertheless, current literature regarding the pathophysiology of IDD remains obscure. Frequently cited as complex multifactorial disease process, IDD results from alterations in the homeostasis of cellular interactions, extracellular matrix and biomechanics [10,15]. Following the initial insult, disc tissues undergo increased cell senescence that is further accelerated by the disc’s relative avascular nature [48]. This renders the disc vulnerable and unable to meet the physiologic demands, ultimately leading to an imbalance between the anabolic and catabolic processes, further causing cellular breakdown [10]. Thus a vicious circle is created. Aging, altered mechanical loading, trauma, metabolic disturbances, and genetics have been associated with IDD [6]; however, the role of obesity has been a topic of debate. Till now, research has solely been focused on its mechanical effects, with little work regarding its metabolic/inflammatory impacts on the disc tissue [18]. The results from the present study indicate that obesity exerts important inflammatory effects on the homeostasis of intervertebral discs that are mediated via adipokines, mainly leptin and adiponectin. It is essential to recognize that further understanding the role of leptin and other adipokines in the pathophysiology of IDD may provide novel avenues for treatment targets, which may potentially halt or reverse IDD.

### 4.1. Presence of Adipokine and Adipokine-Receptors in Intervertebral Discs

Adipokines mediate their effects by binding to their receptors. Current evidence suggests that adipokine receptors are present in both the annulus and the nucleus of the intervertebral disc. In human studies, Koerner et al. reported increased levels of IL-4, IL-5, IL-6, M-CSF, TNF-β, EGF, IGF-1, angiogenin and leptin in the posterior annulus compared to the anterior annulus of degenerative discs [27]. The clinical consequence of this requires further study. Similarly, Gruber et al. immunostained human annulus tissue and showed the presence of intracellular leptin and leptin receptors in annulus cells [29]. Although these results indicate the presence of leptin and its receptor in human disc tissue, however, the authors fail to characterize its isoform. It is known that leptin receptors have different isoforms with the long form (OBRb) being primarily involved in active signaling [49,50]. Therefore, to determine the presence of functional leptin receptors in the disc tissue, Zhao et al. immunostained herniated disc tissues and showed that leptin and functional leptin receptor-positive cells were present in cell clusters and proliferating fibrocartilaginous areas [37]. Likewise, Li et al. reported the expression of mRNA and proteins of OBRb and OBRa in human nucleus tissue and cells [34]. Work on adiponectin was conducted by Terashima et al., who reported the expression of AdipoR1 and AdipoR2 receptors in both human and rat intervertebral disc tissues that was inversely related to disease severity [40]. Likewise, Yuan et al. reported adiponectin levels to be downregulated, while AdipoR1 and AdipoR2 receptor expression to be upregulated in patients with degenerated intervertebral disc tissue compared to healthy controls [39]. This was in contrast to the results reported by Khabour et al., who compared plasma adiponectin levels in patients with degenerative lumbar discs with healthy controls and found that adiponectin levels were elevated in patients with IDD [30]. Since adiponectin is known to possess anti-inflammatory properties [51], one can assume that adiponectin level should be lower in IDD. This disparity could be due to the unadjusted analysis the authors reported, without controlling for body mass index (BMI) or waist–hip ratio between the two groups. Work on resistin by Li et al. found the expression of resistin in degenerated disc tissue [42]. These results indicate that adipokines (leptin, adiponectin and resistin) may play a key role in disc homeostasis; however, their role in disc degeneration needs further exploration.

### 4.2. Effects of Adipokine Treatment on Cellular Proteome

Normal disc cells maintain their matrix structure via a delicate balance between the anabolic and catabolic processes [1,6]. Aging discs show up-regulation of catabolic proteins induced by pro-inflammatory cytokines. Similar results were reported by studies in the present review. In a set of in vitro experiments conducted by Li et al., leptin treatment of nucleus cells from patients with degenerative discs (i) showed proliferative effects and cytoskeletal reorganization [32,33,37]; (ii) induced cyclin D1 expression [33]; (iii) induced F-actin formation and increased β-actin expression [34]; and (iv) inhibited aggrecan expression [35]. These proteonomic changes can affect autophagy, a critical process maintaining homeostasis of intervertebral tissue. Zhang et al. reported that leptin-treated human nucleus cells showed decreased apoptosis rate [38]. This was reversed by treating the nucleus tissue with a leptin inhibitor. Likewise, in animal experiments, Miao et al. reported that leptin-treated nucleus cells exhibited increased mRNA expression levels of metalloproteinase (MMP-1, MMP-13), ADAMTS-4 and ADAMTS-5 [36]. In a study by Li et al., the authors reported that resistin treatment significantly increased the expression of chemokine ligand 4 (CCL4) which promoted macrophage migration in degenerated nucleus cells [42]. These results highlight the pro-inflammatory effects of leptin and resistin on intervertebral disc tissues. In contrast, adiponectin treatment of disc tissue had anti-inflammatory effects, wherein Yuan et al. reported that TNF-α production by degenerated nucleus cells was downregulated by adiponectin administration [39]. Similar results were reported by Terashima et al. These findings highlight that adipokines can modulate the cellular proteome of nucleus cells via multiple signaling pathways and protein expression, activating either a pro- or anti-inflammatory cascade of events. Exploration of these pathways could better elucidate the role of these adipokines as potential target of key pathway points for pharmacologic intervention.

### 4.3. Adipokine Pathways/Signaling

**Figure 3:**
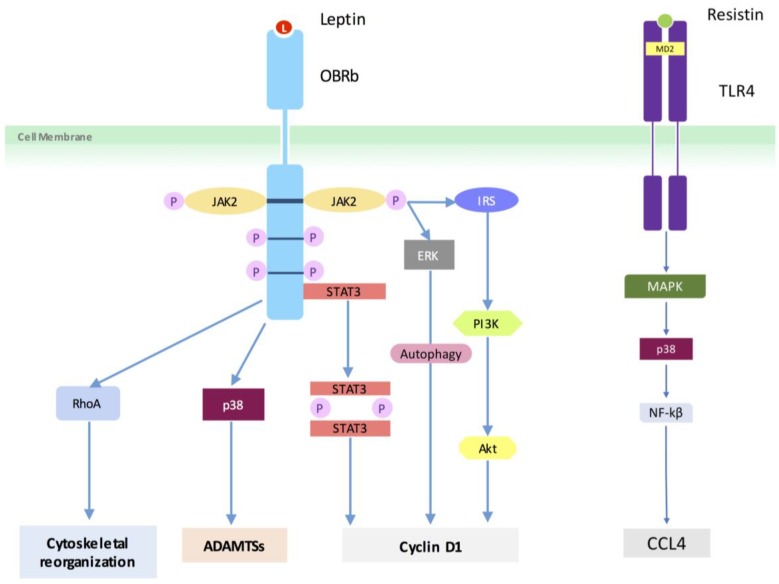
Potential leptin and resistin signaling pathways involved in human intervertebral disc degeneration. Insulin receptor substrate (IRS).

Reports have consistently shown that leptin induces its inflammatory effects by binding to the OBRb receptor subsequently activating several signaling pathways (viz. JAK2/STAT3, IRS/PI3K and MAPK), which are terminated by the induction of suppressor of cytokine signaling 3 (SOCS3) [49,50]. Similar pathway signaling was reported from the results of the present review (Figure 3). In human studies, leptin activated the RhoA/ROCK/LIMK/cofilin-2 cascade to induce cytoskeleton reorganization in nucleus cells [32]. Leptin also induced nucleus cell proliferation and cyclin D1 expression via activation of JAK/STAT3, PI3K/Akt or MEK/ERK signaling [34]. Furthermore, leptin time-dependently increased ADAMTS-4 and ADAMTS-5 protein expression via activation of p38-MAPK pathway, reducing aggrecan protein expression in human nucleus cells [35]. Autophagy of human degenerative nucleus cells was controlled by leptin through the phosphorylated Erk1/2 signal [38]. Similarly, resistin via the TLR4 receptor increased the expression of CCL4 through p38-MAPK and NF-kappaβ signaling in nucleus cells [42]. In rat models, Leptin promoted catabolic metabolism in the rat nucleus cells via the MAPK and JAK2/STAT3 pathways [36].

### 4.4. Adipokine in Other Fibrocartilaginous Diseases

Advances in understanding the role of adipokines in inflammation and fibrocartilaginous disorders are being made. However, it should be noted that the multifaceted functions adipokines play in regard to pathophysiology of various fibrocartilaginous diseases are still not fully understood. Many authors have reported that adipokines can exert both pro- or anti-inflammatory properties. This bidirectional nature of these protein molecules might be influenced by their hosts pathology, genetics, environment, etc. Therefore, reviewing their entire inflammatory effects is out of scope of this current review. However, a brief outline discussing their key properties has been presented. *Leptin*: It is generally considered to be pro-inflammatory adipokine [18]. In OA, leptin has been shown to stimulate chondrocytes to secrete higher levels of cartilage degradation mediators [51]. Similar effects have been reported in patients with rheumatoid arthritis as well [52,53]. *Resistin*: Work regarding the role of resistin fibrocartilaginous diseases is sparse. Current evidence suggests that resistin instigates direct effects on cartilage matrix by increasing cytokine production [19]. It also stimulates proteoglycan degradation, while inhibiting the production of type II collagen [24]. *Visfatin*: It affects the expression of chondrocyte-specific genes involved in extracellular matrix formation. It increases MMP activity and NO production, as well as proteoglycan release in OA cartilage [18]. *Adiponectin*: It has been largely reported to have anti-inflammatory effects. In OA the anti-inflammatory properties of adiponectin are well reported [51].

Despite a broad methodology, the results of this study indicate that little work has been done on this subject, as only 15 studies fulfilled the inclusion criteria. Furthermore, studies were largely being limited to leptin with little work evaluating other adipokines (adiponectin, resistin, adipsin, etc.) that are now actively being investigated in other musculoskeletal diseases [19,27,54]. Although most of the tissue samples collected were from surgical patients, there was a general lack of correlation between the outcome variable and clinical aspects of the IDD (i.e., LBP). The present study is also limited by publication bias and the inherent weaknesses of the studies assessed. Nevertheless, all studies addressed clearly defined hypotheses. Therefore, the results of this review precisely provide the current state of evidence regarding this topic and will form a foundation for future studies.

## 5. Conclusions

Based on the available evidence, in addition to its mechanical effects, obesity does play a key metabolic/inflammatory role in the development of IDD. Adipose tissue can induce a state of systemic inflammation via secretion of pro-inflammatory adipokines, namely leptin. Leptin can act as a mediator by modulating the cellular proteome via different signaling pathways. Conversely, adiponectin was reported to exert anti-inflammatory effects in diseased disc tissues. Further clinical and translational work is required to better understand these molecular pathways activated by adipokine signaling, which may lead to the discovery of potential diagnostic and therapeutic targets.

## Figures and Tables

**Table 1:** Final studies with authors, year of publication, design and key findings.

Authors	Year	Study Design	Key Findings	Species	Sample
Ding et al. [28]	2013	MAPK pathway analyzed using PCR, Western blot and IHC.	p38 and ERK1/2 signalling plays a distinct role in leptin-induced AF cells terminal differentiation.	Rat	AF
Gruber et al. [29]	2007	Disc tissue from *n* = 7 young (normal); *n* = 29 adults (degenerative) examined.	Leptin and its receptor are present in human annulus cells.	Human	AF
Khabour et al. [30]	2014	*n* = 168 LDD patients and *n* = 122 healthy controls were genotyped for rs266729 and rs2241766 SNPs and measured for plasma levels of adiponectin.	Adiponectin was elevated in patients with LDD. However, SNPs in the ADIPOQ gene were not associated with LDD.	Human	Plasma
Koerner et al. [31]	2014	Degenerative (*n* = 7) and normal disc tissue (*n* = 2) was separated into anterior and posterior AF. Expression levels of 42 cytokines were determined and compared between anterior vs. posterior AF.	The posterior AF expressed increased levels of IL-4, IL-5, IL-6, M-CSF, TNF-β, EGF, IGF 1, angiogenin and leptin compared with the anterior AF in patients with degenerative discs.	Human	AF
Li et al. [32]	2014	NP cells isolated from *n* = 7 patients, treated with leptin. RhoA signalling in NP cells was determined. Protein expression of LIMK1 and cofilin-2 was analyzed. F-actin cytoskeletal reorganization was assessed.	Leptin activated the RhoA/ROCK/LIMK/cofilin-2 cascade to induce cytoskeleton reorganization in NP cells.	Human	NP
Li et al. [33]	2012	Effects of leptin on the proliferation of primary cultured human NP cells (*n* = 8) and the underlying mechanism.	Leptin induced human NP cell proliferation and cyclin D1 expression via activation of JAK/STAT3, PI3K/Akt or MEK/ERK signaling.	Human	NP
Li et al. [34]	2013	Do NP tissues and cells express leptin receptors (OBRa and OBRb) and whether leptin affects the organization and expression of major cytoskeletal elements in NP cells (*n* = 45).	mRNA and proteins of OBRa and OBRb were expressed in all NP tissues and cells, and OBRb expression was correlated with body weight. Increased expression of beta-actin, vimentin and reorganization of F-actin were evident in leptin-stimulated NP cells.	Human	NP
Li et al. [35]	2014	Effects of leptin on the expression of aggrecan and ADAMTSs in primary human NP cells (*n* = 4).	Leptin induced p38 to upregulate ADAMTSs and thereby promoting aggrecan degradation in human NP cells.	Human	NP
Li et al. [42]	2017	Resistin and CCL4 expression measured in degenerated human NP tissue. TLR-4, p38-MAPK, and NF-kappaβ signaling pathways studied.	Expression of resistin and CCL4 was elevated in degenerated NP tissue. Resistin via TLR4 receptor increased the expression of CCL4 through p38-MAPK and NF-kappaβ signaling pathways.	Human	NP
Liu et al. [41]	2016	Transcriptional activity, gene expression, and protein levels of ADAMTS-5 were measured in resistin-exposed NP cells along with detection of activation of p38 MAPK.	p38-MAPK signaling pathway was activated after exposure to resistin. p38 inhibitor decreased the upregulation of ADAMTS-5 by resistin.	Rat	NP
Miao et al. [36]	2015	Effects of leptin on the expression of degeneration-associated genes in *n* = 30 rat NP cells, and its possible mechanism.	Leptin promoted catabolic metabolism in the rat NP cells via the MAPK and JAK2/STAT3 pathways.	Rat	NP
Terashima et al. [40]	2016	Adiponectin and adiponectin receptors AdipoR1 and AdipoR2 were detected in disc tissue (*n* = 4, Humans) and (*n* = 21, Rats). IL-1beta and/or adiponectin-treated rat NP and AF tissues were evaluated for mRNA expression of TNF-alpha and IL-6.	AdipoR1 and AdipoR2 were widely expressed in both human and rat IVD tissues, were inversely related to disease severity. TNF-alpha expression in the IL-1beta + Ad group was significantly lower than that in the IL-1beta group in both NP and AF cells.	Human, Rat	NP, AF
Yuan et al. [39]	2018	Examined the expression levels of and effect of adiponectin on TNF-alpha in IVD tissues and isolated NP cells.	Adiponectin levels were downregulated, while AdipoR1 and AdipoR2 expression was upregulated in degenerated IVD tissues and NP cells compared to healthy controls. TNF-alpha production by degenerated NP cells was downregulated by adiponectin administration.	Human	NP
Zhang et al. [38]	2018	Human degenerative NP cells were extracted and cultured, then treated with leptin, leptin inhibitor and leptin neutralizing antibody and expressions of LC3 II/I, Beclin-1 were studied and change of apoptosis rate was detected. Leptin/bafilomycin A and (PI3K)/(MEK) inhibitor-treated cells were used to detect the expressions of LC3II/I, cleaved caspase 3, apoptosis rate, Akt and Erk1/2 signal pathway.	Leptin-treated cells showed increased expressions of LC3II/I and Beclin-1 and decreased apoptosis rate. Leptin inhibitor or neutralizing antibody showed the opposite results. Bafilomycin A increased the expression of LC3II/I and apoptosis rate. Inhibition of Akt phosphorylation was partially offset by leptin while inhibition of Erk1/2 phosphorylation was not.	Human	NP
Zhao et al. [37]	2008	Determined the expression of leptin and its functional receptor in human herniated disc tissues (*n* = 45), and to elucidate whether leptin can stimulate rat NP cells to proliferate in vitro.	Disc cells express leptin and its functional receptor. Leptin stimulated proliferation of disc cells in vitro.	Human, Rat	NP

Janus Kinase 2 (JAK2), Signal transducer and activator of transcription (STAT), Mitogen activating protein kinase (MAPK), 3-(4,5-dimethylthiazol-2-yl)-2,5-diphenyltetrazolium bromide (MTT), proliferation cell nuclear antigen (PCNA), a disintegrin and metalloproteinase with thrombospondin motifs (ADAMTS), Leptin Receptor Long Form (OBRb), Ras homolog gene family member A (RhoA), matrix metalloproteinase (MMP), nucleus pulposus (NP), annulus fibrosus (AF), tumor necrosis factor-alpha (TNF-α), tumor necrosis factor-beta (TNF-β), interleukin (IL), adiponectin receptor 1 (adipoR1), adiponectin receptor 2 (adipoR2), extracellular regulated protein kinases 1/2 (Erk1/2), phosphatidylinositol 3-kinase (PI3K), extracellular regulated protein kinases (MEK), light chain 3 (LC3), chemokine ligand 4 (CCL4), Toll-like receptor 4 (TLR-4), nuclear factor-kappa beta (NF-κβ), Intervertebral disc (IVD), Lumbar disc degeneration (LDD), Macrophage colony-stimulating factor (M-CSF), epidermal growth factor (EGF), Insulin-like growth factor 1 (IGF 1), Single nucleotide polymorphisms (SNPs), LIM domain kinase 1 (LIMK1), Rho-associated protein kinase (ROCK), Enzyme-linked immunosorbent assay (ELISA), Immunohistochemistry (IHC), Polymerase chain reaction (PCR).
